# New Strategies on Green Synthesis of Dimethyl Carbonate from Carbon Dioxide and Methanol over Oxide Composites

**DOI:** 10.3390/molecules27175417

**Published:** 2022-08-24

**Authors:** Yifei Zhang, Muhammad Shoaib Khalid, Meng Wang, Gao Li

**Affiliations:** 1 Institute of Catalysis for Energy and Environment, College of Chemistry and Chemical Engineering, Shenyang Normal University, Shenyang 110034, China; 2 State Key Laboratory of Catalysis, Dalian Institute of Chemical Physics, Chinese Academy of Sciences, Dalian 116023, China; 3 Key Laboratory of Biofuels and Biochemical Engineering, SINOPEC Dalian Research Institute of Petroleum and Petro-Chemicals, Dalian 116045, China

**Keywords:** green synthesis of dimethyl carbonate, carbon dioxide, methanol, Zr/Ce-based oxides, Cu-based nanoparticle

## Abstract

Dimethyl carbonate is a generally used chemical substance which is environmentally sustainable in nature and used in a range of industrial applications as intermediate. Although various methods, including methanol phosgenation, transesterification and oxidative carbonylation of methanol, have been developed for large-scale industrial production of DMC, they are expensive, unsafe and use noxious raw materials. Green production of DMC from CO_2_ and methanol is the most appropriate and eco-friendly method. Numerous catalysts were studied and tested in this regard. The issues of low yield and difficulty in tests have not been resolved fundamentally, which is caused by the inherent problems of the synthetic pathway and limitations imposed by thermodynamics. Electron-assisted activation of CO_2_ and membrane reactors which can separate products in real-time giving a maximum yield of DMC are also being used in the quest to find more effective production method. In this review paper, we deeply addressed green production methods of DMC using Zr/Ce/Cu-based nanocomposites as catalysts. Moreover, the relationship between the structure and activity of catalysts, catalytic mechanisms, molecular activation and active sites identification of catalysts are also discussed.

## 1. Introduction

DMC is an eco-friendly chemical compound and intermediate which is versatilely used as a raw material in polycarbonate manufacturing [1,2] and in the production of dimethyl phenol (C_8_H_10_O) [3]. The high needs of PC resulted in its increased production reaching up to 5.15 million tons in 2015. Moreover, DMC is also being extensively used as an electrolyte in Lithium-ion batteries by virtue of the high value of its dielectric constant [4]. It is replacing methyl tertbutyl ether (MTBE) as an additive for fuel oil owing to its non-toxicity, rapid biodegradable quality, outstanding gasoline/water distribution coefficient, excessive oxygen content of 53% and high-octane value of 105 [5]. The suitable amount of DMC in diesel oil results in the decreased production of soot particles, thus reducing pollution [6,7]. Added to this, eco-unfriendly phosgene and dimethyl sulfate are being replaced by eco-friendly DMC-assisted methyl/carbonyl groups which are eventually being used as reagents in methylation and carbonyl reactions [8]. Nowadays, the main standard to produce DMC is an industrial process approach known as TTF (Dutch Title Transfer Facility). Ethylene oxide- and propylene oxide-like compounds are not suitable to be used as additives for fuel oil because their reactants are costly; the chlorohydrination process overcomes this through eco-friendly harmfulness [9]. Therefore, producing DMC by using inexpensive and eco-friendly methods will reimburse the market gap by replacing MTBE.

**Figure 1:**
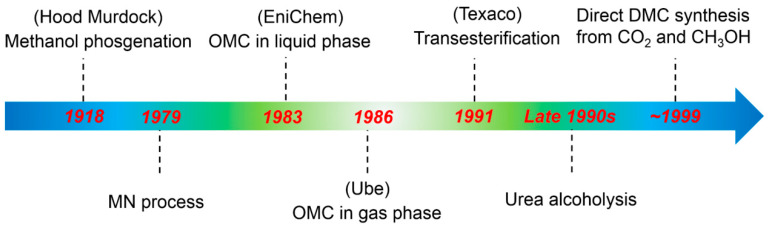
Evolution of DMC synthesis.

Until now, phosgenation, TTF and liquid-phase MeOH-oxidative CBN have been applied for the industrial production of DMC [10] (Figure 1). Later, phosgenation was quitted because of its use of extremely toxic phosgene as a raw material. TTF and liquid-phase MeOH-oxidative CBN methods are still being used at the fabrication level in industries, but have some serious drawbacks, e.g., the TTF method is expensive and produces large amounts of effluent water [11], whereas in the latter, the water formed initiates catalyst deactivation [12,13]. Therefore, a lot of research needs to be conducted for the clean, green and technically improved synthesis of DMC.

Several aspects such as synthesis methods, the selection of raw materials, catalysis and industrial implementation have been discussed in previous review papers on the synthesis of DMC [6,14,15]. This review paper focuses on DMC synthesis methods and their applications while the pros and cons of different synthesis methods are also critically analyzed. The current state of industrial DMC synthesis has been well-documented in previous reviews and it is not included in this one [16,17,18]. Therefore, we will only discuss established green synthesis methods of DMC using CO_2_ and methanol. Some crucial aspects including reaction mechanism, reaction conditions, catalyst characteristics as well as industrial implementation will be considered. Finally, we will present some future recommendations.

## 2. Developed Methods for Green DMC Synthesis

Synthesizing useful chemical products from waste CO_2_ is known as the “green” chemical method [19]. Nowadays, a great deal of research is being conducted in this regard [20,21]. As CO_2_ is thermodynamically stable and kinetically inert, its activation has always been challenging [22]. The issue of thermodynamic inertness can be solved by controlling the required dehydration rate and pressurizing CO_2_. Two particular agents, i.e., non-recyclable (dicyclohexylcarbodiimide; orthoesters) and recyclable (molecular sieves; acetals), are used to increase the yield of DMC.

### 2.1. Thermodynamics

The thermodynamic data of DMC were initially measured by using the increment theory of the Benson’s group. As reported in the literature, many basic physical quantities of thermodynamics were used in its calculations [23]. The production of DMC using CO_2_ and methanol (Equation (1)) is achievable because of the reaction’s thermodynamics.

(1) $$2{\mathrm{CH}}_{3}{\mathrm{OH}+\mathrm{CO}}_{2}(g)\;\to DMC+H_{2}O$$

Standard molar reaction enthalpy:

(2) $$\Delta H_{r}^{0}(298k)=\Delta H_{f}^{0}(DMC)+\Delta H_{f}^{0}(H_{2}O)-2\Delta H_{f}^{0}(CH_{3}OH)-\Delta H_{f}^{0}(CO_{2})=-17kJ/mol$$

As per calculations, the reaction is exothermic with a small amount of heat release [24], and elevated reaction temperatures are harmful to the reaction, as inferred by the following equation:

(3) $$\Delta H_{r}^{0}(T)=-2.04\times 10^{4}+4.63T+25.78\times 10^{-3}T^{2}-18.03\times 10^{-6}T^{3}J/mol$$

Consequently, the heat liberated reduces remarkably with the rising temperature. In a definite range, decreasing the temperature will raise the conversion. It can be calculated according to:

(4) $$\Delta G_{r}^{0}(298K)=\Delta H_{f}^{0}(298K)-T\Delta S_{f}^{0}(298K)=25kJ/mol$$

(5) $$\mathrm{Ln}K^{0}(298K)=\frac{\Delta G_{r}^{0}(298K)}{-RT}=-10$$

The standard molar Gibbs function is calculated as:

(6) $$\Delta G_{r}^{0}(T)=-20.37\times 10^{3}+185.98T-4.66T\mathrm{lnT}-25.77\times 10^{-3}T^{2}+8.98\times 10^{-6}T^{3}J/mol$$

Then, the equilibrium constant Ink^0^ can be found out as:

(7) $${\mathrm{LnK}}^{0}(T)=-22.37+2.45\times 10^{3}T^{-1}+0.56\mathrm{lnT}+3.1\times 10^{-3}T-1.08\times 10^{-6}T^{2}$$

Or the constant K^0^ is indicated as:

(8) $$K^{0}=\frac{C^{2}}{{4(1-c)}^{3}}/\frac{p}{p^{0}\left(3-c\right)}$$

where, p and c are the equilibrium pressure and CO_2_ equilibrium conversion.

The CO_2_ equilibrium conversion is around 10% at a temperature of 273 K and 10 MPa pressure. By carefully regulating the reaction pressure, the equilibrium conversion can be improved. Generally, the yield is increased by decreasing the temperature and raising the pressure. Contrarily, a huge amount of CO_2_ is required to enhance the MeOH conversion. On the whole, the thermodynamic studies indicate that the green production of DMC is achievable under proper conditions.

### 2.2. Thermal Catalytic Synthesis

**Figure 2:**
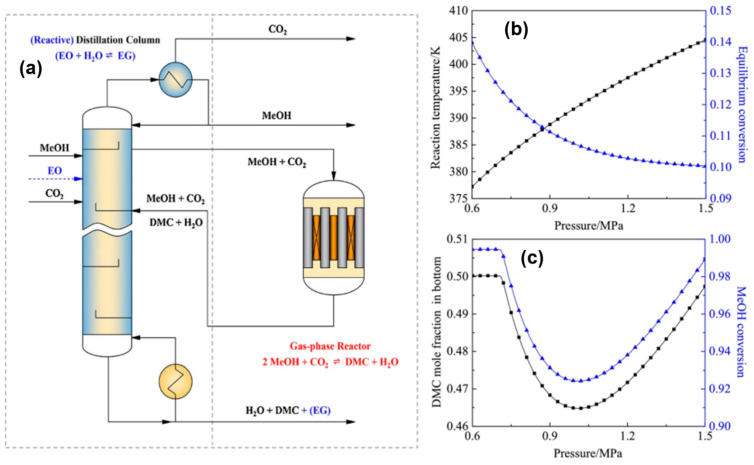
(**a**) Reactor with in situ hydration for green synthesis of DMC. Effect of pressure on (**b**) reaction and (**c**) reactive distillation. Reproduced with permission from Ref. [27]. Copyright 2018 Elsevier B.V.

Green DMC synthesis utilizing membrane reactors was studied in 2003 by Zhong et al. [25]. Membranes of three different types—inorganic silica, hybrid polyimide–silica and hybrid polyimide–titania—were used. In terms of catalytic effectiveness, the polyimide–silica hybrid membrane reactor surpassed the silica and polyimide–titania membrane reactors. At 130 °C and 0.4 MPa pressure in the polyimide–silica membrane reactor, Cu-KF/MgSiO_x_ provided 9.2% MeOH conversion and 96.0% DMC selectivity. Under the same operating conditions, the Cu-KF/MgSiO_x_ catalyst produced 6.5% MeOH conversion at 90% DMC selectivity in comparison to traditional reactors. In comparison to traditional reactors, all three membrane reactors produced more DMC. While the DMC selectivity rose just a little under ideal reaction conditions, the MeOH conversion increased dramatically. Be aware that when the reaction temperature rises, the activity increases but the DMC selectivity falls, shortening the catalyst’s life. For the selective removal of water by-products in the membrane reactors, Wang and colleagues [26] used hydrophilic Sodalite and Linde Type-A (LTA) membranes. The H_2_O/CH_3_OH combination’s pervaporation split-up aspect was improved by the LTA membrane when utilized at room temperature, going from 2.8 to 7.4. Furthermore, the H_2_O/DMC mixtures that the LTA membrane successfully separated showed selectivity values prior to (800) and (1000) ion exchanges. The new fixed-bed reactor (Figure 2a) with the side reactor for DMC synthesis was created by Qi and colleagues in 2018 [27]. The only process that occurred in the innovative reactor was the in situ hydration of EO. A distillation column was used for separation, and a gas-phase side reactor was used for EO hydration. At 0.6 MPa pressure and 140 °C, this accelerated reactor could provide an MeOH conversion of 99.5% with maximal DMC selectivity (Figure 2b,c). The system was treated with EO to improve the elimination of H_2_O. To boost the catalytic activity in the green synthesis of DMC, the reactor must be remarkably upgraded.

Recently, the thermodynamics and environmentally friendly synthesis of DMC over a yttrium oxide catalyst (Y_2_O_3_) was investigated [8]. Above 60 °C, the reaction started spontaneously. Because of its mild acidic and basic sites that may effectively activate CO_2_ and CH_3_OH, the Y_2_O_3_-750 catalyst beat the other catalysts in terms of catalytic efficiency. The DMC yield rose monotonically as the amount of moderately acidic sites increased. At 90 °C and 8 MPa pressure, the DMC yield and CH_3_OH conversion were at their maximum levels.

### 2.3. Zr-Based Catalysts

The reductive oxides have been well-documented in the redox reactions [28,29,30,31,32,33]. Zirconia is a top pick among potential catalysts for the green DMC production. Based on in situ DRIFTS experiments, a chemical pathway was hypothesized where MeOH adsorption occurs initially on O_2_ atoms of Zr^+^ cations [34]. The methoxy intermediate CH_3_O- and protons that are created by the dissociation of adsorbed MeOH react with the hydroxyl surface groups of zirconia to create water molecules. Following this interaction between CO_2_ and the O atoms in the Zr-O groups, which results in the formation of the methyl carbonate intermediate, DMC is created using a procedure that is identical to that of CO_2_ by adding another MeOH molecule to the intermediate [34]. Recent research by Li et al. suggested that oxygen vacancies (Ov) encourage the synthesis of green DMC over Zr-doped CeO_2_ nanorods (NRs). They utilized a hydrothermal technique to produce various Zr-doped CeO_2_ NRs and looked into the influence of the Zr doping level on the lattice structure and microstructure, specifically the Ov density together with catalytic activity. The catalytic activity was shown to be correlated with the surface Ov concentration using Zr_0.1_Ce NRs with the highest concentration of Ov defects for the synthesis of DMC. A fresh method for creating effective CeO_2_-based catalysts was presented in this study. Adsorbed CO_2_ is first activated by an Ov site through a Lewis acid–base reaction close to the Ov site.

Recently, Liu et al. [9] created a highly stable and selective ZrO_2_-Al_2_O_3_ co-precipitation catalyst for the reactive distillation and DMC synthesis. With a focus on the acid–base balance and its connection to catalytic performance, the impact of the aluminum concentration on structural characteristics and catalyst acid–base efficiency was examined [35]. A reactive distillation column was used to assess the catalytic effectiveness. While the Zr_1−x_Al_x_O_y_ catalysts offered outstanding TTF efficiency for the synthesis of propylene carbonate (PC) and MeOH, the typical amphoteric compounds, including ZrO_2_, Al_2_O_3_ and ZrO_2_ + Al_2_O_3_, provided low conversion and selectivity for the formation of DMC [5,36]. The Zr_0.5_Al_0.5_O_y_ catalyst had the highest PC conversion of 98.1% at 99.9% DMC selectivity among the catalysts. The fact that the DMC production rose linearly with the quantity of both weak acid and strong base centers shows that the PC and MeOH TTF can be efficiently catalyzed by the catalyst containing acid–base sites. Because of the surface-specific sites on the catalysts, MeOH was able to split into CH_3_O-(a) and H, which then attacked the carbonyl group on PC to produce an intermediate. This intermediate then reacted with H^+^ to form 2-hydroxypropyl methyl carbonate, which in turn reacted with another CH_3_O^−^ to produce DMC [37]. The best possible efficiency of this reaction pathway depends on the catalyst’s capacity to dissociate MeOH.

**Figure 3:**
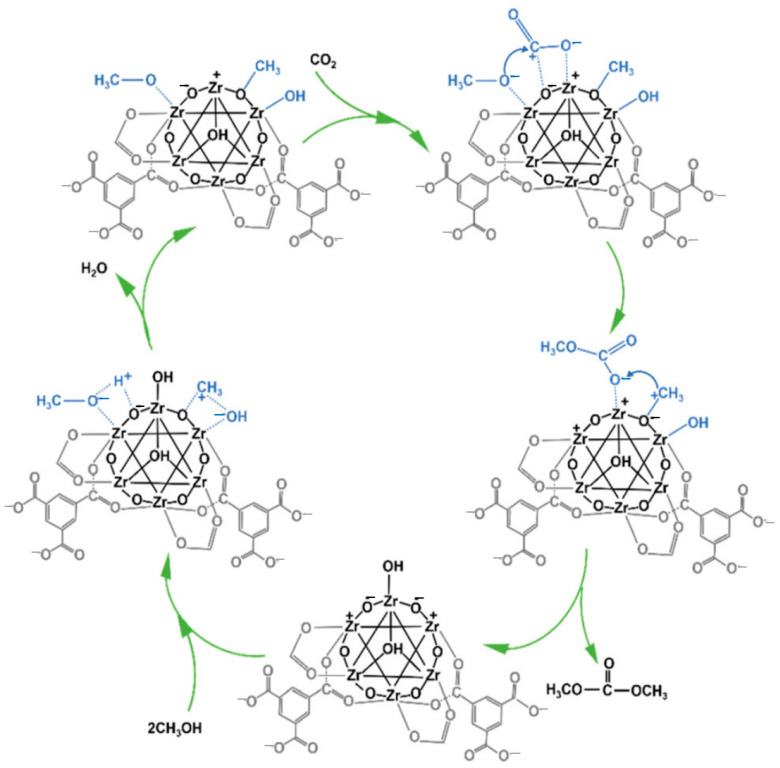
Proposed reaction mechanism for the direct synthesis of DMC from CO_2_ and CH_3_OH over MOF-808. Reproduced with permission from Ref. [40]. Copyright 2018 Elsevier B.V.

In the meantime, the title reaction was carried out by the research team of Xuan et al. using Zr-based MOFs, such UiO-66 and MOF-808 [38]. By adjusting the number of ligands employed, two series of MOF catalysts were created: trifluoroacetic acid (TFA) for UiO-66 and 1, 3,5-benzenetricarboxylic acid (BTC) for MOF-808. It was found that the surface area, pore size and acid–base site could all be altered by changing the number of ligands employed. Greater DMC yields were achieved with the solids synthesized with 240 mmol TFA (1479 m^2^ g^−1^ of surface area and 9.8 mmol g_cat_.^−1^ of total acid–base sites) and 32 mmol BTC (1373 m^2^ g^−1^ and 8.3 mmol g_cat_^−1^) than with other solids synthesized with different ligand amounts (0.084% and 0.12%, respectively). Despite UiO-66′s bigger surface area and larger amount of acid–base sites, MOF-808 demonstrated higher catalytic activity. This is explained by the fact that MOF-808 has larger micropores than UiO-66, increasing the accessibility of reactants to the active sites housed within the micropores. Figure 3 illustrates the suggested reaction process for this kind of catalyst in which the acidic sites of MOF-808-adsorbent 4′s CH_3_OH first make Zr-OCH_3_, which releases the hydrogen atom, by forming a strong bond with exposed Zr^4+^ at the Zr_6_ node. The rapid interaction between the hydrogen atom and the terminal hydroxyl (Zr-OH) produces H_2_O. After CH_3_OH adsorption, there is an increase in the intensity of the hydroxyl group at the Zr6 node (band at 3700–3500 cm^−1^), suggesting that the generated H_2_O may align with the exposed Zr^4+^ to make Zr-OH_2_ [39]. The water that has been produced during the actual reaction can be removed by reacting with trimethoxymethane. The ability of the acidic sites (Zr^4+^) to trigger the conversion of CH_3_OH into the methyl cation (CH^3+^) is also noteworthy. The bridging methoxy (Zr-(OCH_3_)-Zr) moiety can then be produced by adsorbing the generated CH^3+^ on the basic site (unsaturated O_2_-in Zr-O-Zr or Zr-O) of the Zr_6_ node. Thus, the terminal hydroxyl (Zr-OH) could be formed when the hydroxyl freed from CH_3_OH adsorbs on the acidic site of Zr^4+^. After being exposed to CO_2_, the basic sites in MOF-808-4 absorb CO_2_, which inserts into Zr-OCH_3_ to produce the intermediate Zr-OCOOCH_3_. DMC is created when Zr-OCOOCH_3_ interacts with CH^3+^ that has been liberated from Zr-(OCH_3_)-Zr.

It is important to consider the possibility of CO_2_ interacting with the catalyst during the synthesis of DMC. Based on the reaction mechanism outlined above, CO_2_ can be activated to create bidentate bicarbonate species (b-HCO_3_-Zr) and bidentate carbonate species (b-CO_3_-Zr). The b-HCO_3_-Zr interacts with CH_3_OH to create an intermediate methyl carbonate and produce HCO_3_. The interaction of t-Zr-OCH_3_ with CO_2_ produces the methyl carbonate intermediate more quickly than this method does. Therefore, it was proposed that, as shown in Figure 3, the majority of the production of DMC occurs through the interaction of CH^3+^ and methyl carbonate, which is produced when t-ZOCH_3_ reacts with CO_2_ [40].

Using H_3_PW_12_O_40_/Ce_0.1_Ti_0.9_O_2_ as a catalyst, Chiang et al. produced dimethyl carbonate through the carbonation of methanol. The Ov sites affect this reaction’s mechanism. The H_3_PW_12_O_40_/Ce_0.1_Ti_0.9_O_2_ catalyst’s surface is significantly covered in oxygen vacancies as a result of crystal structural flaws. An O atom from a CO_2_ molecule and a hydrogen atom from the interacting MeOH molecule each fill a vacant defect in the suggested mechanism. The development of an unstable intermediate follows the subsequent adsorptive attachment of a further MeOH molecule to a neighboring Ov. The chemical cycle is finally completed when these intermediaries disintegrate, producing DMC and H_2_O that desorb from Ov. In terms of Ov and crystal defects, this method explains the essential basis of the behavior of heteropoly acid catalysts.

### 2.4. Ceria-Based Catalysts

Ceria-based oxide catalysts have attracted a lot of interest because of their promising characteristics, especially the remarkable oxidation-reduction properties and increased O_2_ storage capability [41,42,43,44,45,46,47]. According to earlier findings [48], CeO_2_ has a strong catalytic capacity for dehydration processes, indicating that it might be employed in the synthesis of DMC. CeO_2_, on the other hand, typically has a poor surface area and a short roast life at high temperatures, which can occasionally lead to a quick deactivation rate (less than ten hours) during catalytic processes. To overcome the constraints of CeO_2_, transition metals have been doped into the CeO_2_ lattice to create M_x_Ce_1−x_O_y_ composites. For instance, Zr-doped CeO_2_ nanorods (Zr_x_Ce_1−x_O_2_) have been found to have a higher Ov concentration than pristine CeO_2_, which makes it simpler for CO_2_ to be activated by interacting with the surface Ov to produce the bidentate carbonate intermediate, as proven by in situ FTIR analysis [49]. We recently made the first demonstration of DMC synthesis from CO_2_ and MeOH utilizing monolithic Zn_x_Ce_1−x_O_δ_ catalysts [50]. Zn_x_Ce_1−x_O_δ_ (x: 0–0.20) nanoparticles (NPs) were created utilizing an aqueous-phase co-precipitation procedure. The produced NPs were uniformly coated on honeycomb ceramics, and the green synthesis of DMC was used to assess their catalytic performance. Large surface area, reduced pressure drop and superior heat and mass transfer are just a few benefits that monolithic catalysts can display. Additionally, water produced as a by-product of the reaction can be removed more quickly, preventing the catalyst from deactivating and hydrolyzing, and shifting the equilibrium to the products’ side. This is especially advantageous for reactions such as the synthesis of DMC that are constrained by the thermodynamic equilibrium. The CH_3_OH conversion increased with the catalyst’s Zn content until it reached a maximum, then dropped with more Zn. Of all the monolithic catalysts examined, the monolithic Zn_0.10_Ce_0.90_O_δ_ catalyst demonstrated the best MeOH conversion at 20.5%. When the Zn content and reaction temperature increased, the DMC selectivity gradually dropped. Dimethyl ether (DME), formaldehyde and CO were produced as by-products. When MeOH is dehydrated, DME forms when CH_3_OH is activated, and HCHO forms when CO2′s C–O link is broken [51,52]. To further enhance the reaction conditions, the most active Zn_0.10_Ce_0.90_O_δ_ monolithic catalyst was used. The effect of changing the reaction temperature from 100 to 180 °C revealed a volcano-type curve for the relationship between the MeOH conversion and temperature, which is that the MeOH conversion rises with temperature, reaching a maximum of 20.5% at about 160 °C, then falls, while the selectivity to DMC steadily decreases from 88.7% to 77.5%. Compared to the granular catalyst, the monolithic Zn_0.10_Ce_0.90_O_δ_ composite demonstrated improved durability. Following 48 h on-stream, there was just a slight decline in the CH_3_OH conversion and DMC selectivity, from 20.5% to 19.1% and 82.3% to 81.2%, respectively.

The same group [53] also devised a technique for creating monolithic catalysts for green DMC synthesis by preparing Ti-doped CeO_2_ nanocomposites layered on honeycomb ceramics. The catalytic results show that, at 140 °C, without the use of any dehydration agents, raising the surface Ov concentration through Ti dopants leads to a 24.3% MeOH conversion and a 78.6% DMC selectivity. For 48 h while it was on-stream, the monolithic Ti_0.1_Ce_0.9_O_2_ catalyst demonstrated good stability in the green DMC synthesis. The Ti_0.1_Ce_0.9_O_2_ displayed the highest MeOH conversion at the ideal temperature of approximately 140 °C. The Ti_0.1_Ce_0.9_O_2_ catalytic action is temperature-dependent. The conversion of CH_3_OH was substantially lower with the corresponding particulate catalyst. At 140 °C, the monolithic catalyst’s productivity could reach up to 41.1 mmol_DMC_ g_cat__._^−1^ h^−1^, which is approximately twice as productive as the particulate catalyst’s 22.1 mmol_DMC_ g_cat._^−1^ h^−1^.

**Figure 4:**
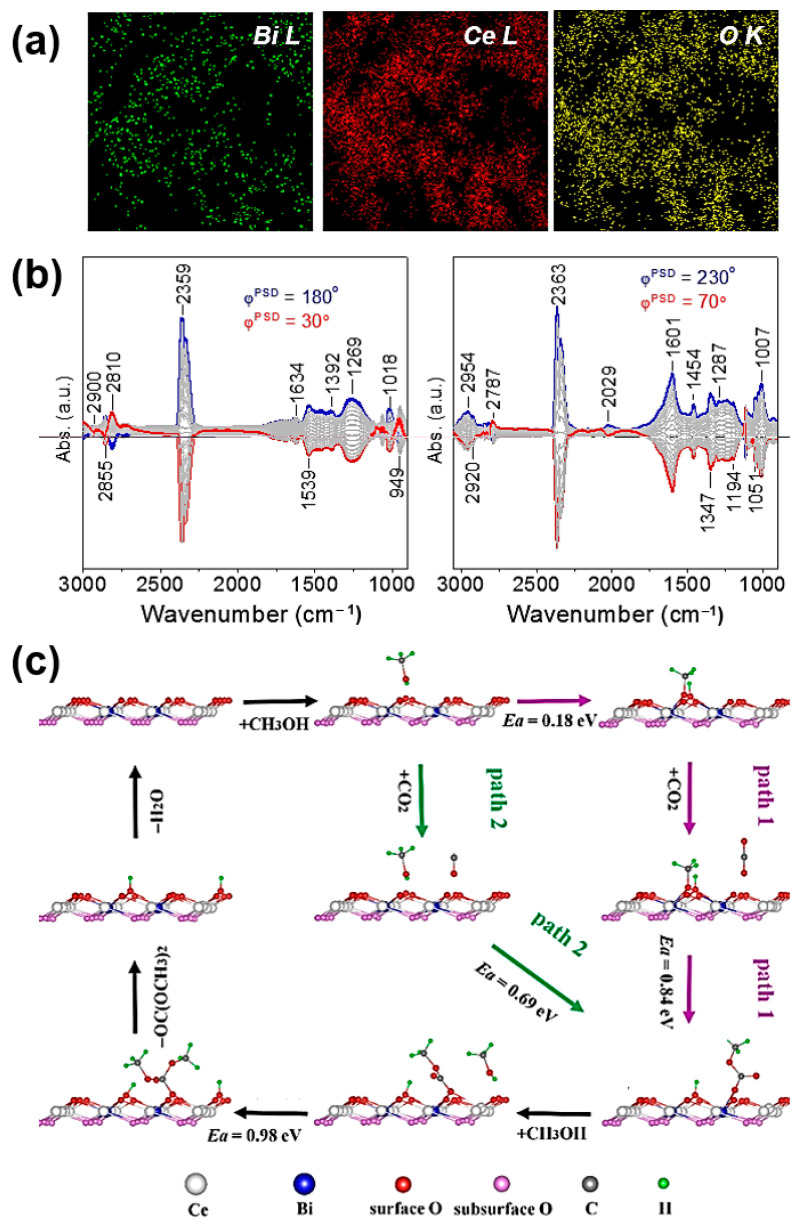
(**a**) TEM image and element mapping of Bi_0.12_Ce_0.88_O_δ_ nanocomposite. (**b**) Phase-domain DRIFT spectra on the CO_2_ adsorption over Bi_x_Ce_1−x_O_δ_ composite: switching between CO_2_ ↔He at 25 °C and CH_3_OH + CO_2_↔He at 140 °C. (**c**) Proposed reaction mechanism over the Bi_x_Ce_1__−x_O_δ_ nanocomposite based on DFT study. Reproduced with permission from Ref. [53]. Copyright 2021 Springer Nature.

Recent research has shown that ceria’s surface Ov concentration and activity in the green synthesis of DMC may be significantly increased by bismuth doping [53]. These catalysts’ elemental mapping reveals that their components are uniformly scattered (Figure 4a). To synthesize monolithic catalysts, the BiCeO_x_ nanocomposites were additionally coated over a ceramic honeycomb (cordierite). A maximum Ov concentration was observed in Bi_0.12_Ce_0.88_O_δ_, which was probed by using electron paramagnetic resonance (EPR), X-ray photoelectron spectroscopy (XPS) and Raman spectroscopy. A gas hourly space velocity of 2880 mL g_cat_^−1^ h^−1^ and 45 h on-stream at 140 °C was achieved by this catalyst, which provided the highest DMC production rate.

According to temperature-programmed desorption (TPD) of methanol CH_3_OH and CO_2_, the Bi-dopants boost the CO_2_ adsorption uptake of ceria and CO_2_ activation plays a vital role in the production of DMC. Although the acidic property of the Bi_x_Ce_1−x_O_y_ composites moderately reduced while raising the concentration of Bi-dopant, the NH_3_-TPD findings do not support a clear association between acidic property and catalytic activity. An in-depth understanding of the species on the catalyst surface was achieved by using in situ DRIFTS studies in combination with modulation excitation spectroscopy and phase-sensitivity detection. Carbonate and bidentate carbonate species made up the majority of the species adsorbed on the oxide surface (Figure 4b). Monodentate methyl carbonate species (CH_3_O-C(=O)-O) were found to be the reaction intermediate under the reaction circumstances (CH_3_OH + CO_2_↔He) and they swiftly reacted with the nearby activated methoxy species to produce the final DMC product (Figure 4b). In the end, first-principle computations confirmed these experimental findings. Two actively possible reaction mechanisms were proposed (Figure 4c) in which the formation of monodentate methyl carbonate by the interaction of CO_2_ with adsorbed methoxy (CH_3_O_a_) is thought to be the rate-determining step in the synthesis of DMC.

Possessing three distinct ceria forms (rods, cubes and octahedra), a series of ceria-supported metal oxide catalysts were latterly created by Rownaghi et al. [12]. In comparison to cubes (CeO_2_(100)) and octahedra (CeO_2_(111)), ceria nanorods exposing (110) and (100), facets demonstrated higher catalytic performance. The highest DMC yield of ~1.6 mmol was given by CeO_2_ nanorod catalysts in comparison to other CeO_2_-supported CoO (1.33 mmol), NiO (1.0 mmol), CaO (0.82 mmol) and CuO catalysts (0.27 mmol). Additionally, after the produced catalysts underwent four sequential reuse cycles, the catalytic activity of CeO_2_ and CoO_x_/CeO_2_ slowly reduced. Moreover, the catalytic effectiveness of CoO_x_/CeO_2_ was investigated using a variety of loadings, and the results showed that the CoO loading had no impact on the DMC yield for metal oxide loadings between 2.5–10 wt%. In summary, equal acid–base site densities in NiOx/CeO_2_ and CeO_2_ led to marginally different DMC yields and catalytic results, although more clarification is needed on the function of the catalyst’s acid–base characteristics.

Darbha et al. [54] assessed the DMC produced with the help of 2-CP (2,5-dimethoxy-4-propylphenethylamin, a dehydrating/water trapping agent). Due to an optimum mix of medium acid–base sites and defect centers, CeO_2_-spindles have shown to be a more effective catalyst than rod-, cube- and irregular-shaped CeO_2_. MeOH was quantitatively converted with approximately 100% selectivity to DMC over the ceria-spindle catalyst. MeOH conversion and DMC yield were proportional to the amount of medium-strength acid–base sites and defect sites found on (111) planes. Methoxide ions were produced by simple sites, whereas CH_3_^+^ ions from MeOH were produced by acid sites. Proper acidic and basic site alteration is required to achieve 100% DMC selectivity. The ideal ratio of these active sites is reportedly present in the ceria-spindle catalyst, increasing conversion and DMC selectivity.

As the number of medium acid and base sites grew, MeOH to DMC conversion and DMC yield also increased. The varying acid–base sites’ strengths in the various ceria catalysts were blamed for the divergence from linearity. The greater temperature at which NH_3_ desorbs from ceria-spindle catalysts than from the other catalysts suggests that Ce-spindles have more potent acid–base sites. The ceria-spindles’ strong catalytic activity is ascribed to their shape, which offers the ideal blend of defect sites and moderate acid–base sites. This study not only confirms numerous earlier findings, but also highlights the crucial importance of moderately strong acid–base sites. By incorporating metal/metal oxides, Urakawa et al. [13] used conventional procedures to change the chemical characteristics of CeO_2_, specifically the acidity/basicity. Numerous studies were conducted on their impacts on the oxygen storage capacity, defect stability, catalyst stability and O atom mobility.

**Figure 5:**
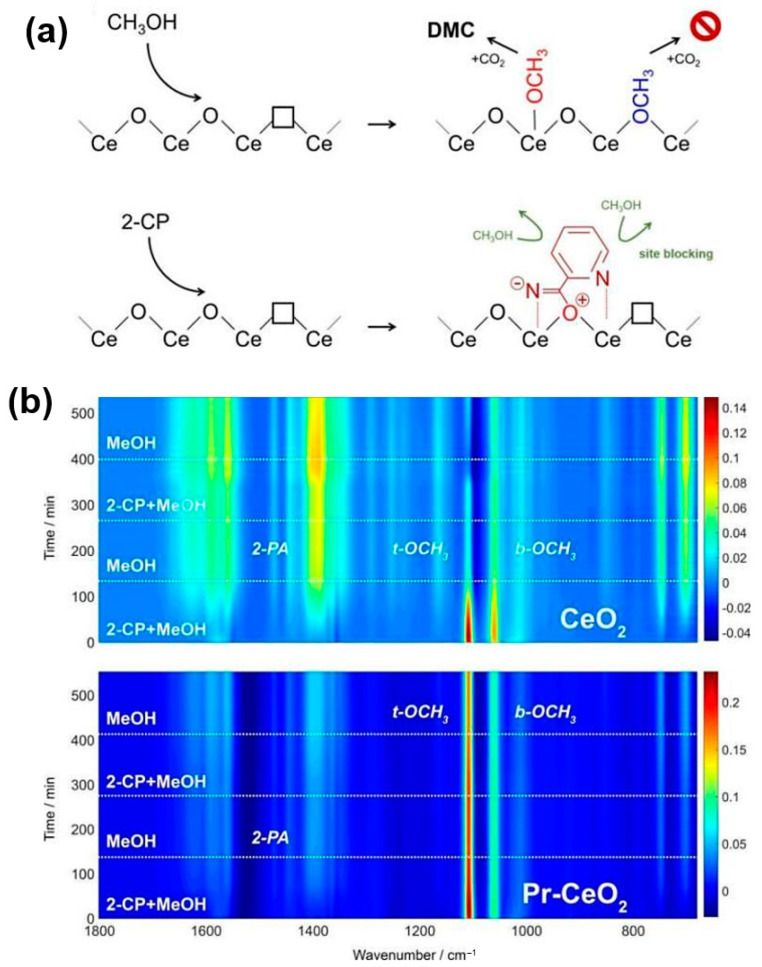
(**a**) Simplified DMC formation over CeO_2_ by the coupling between t-OCH_3_ and CO_2_ with the aid of 2-CP. The hydrolysis of 2-CP on the CeO_2_ surface causes blocking of the sites for MeOH adsorption. (**b**) In situ ATR-IR spectra of the formed species by alternatingly passing the MeOH vapor and mixed MeOH + 2-CP vapor over CeO_2_ and Pr-CeO_2_ at 120 °C. Reproduced with permission from Ref. [13]. Copyright 2018 American Chemical Society.

To slightly alter the acid–base characteristics, the impacts of La [55,56], Gd [57,58] and Pr [59,60,61] promoters on the materials linked to Ov defects/CeO_2_ mobility were thoroughly investigated. Under specified reaction circumstances (such as at 120 °C and 30 bar), the deactivation state of the catalysts was visually inspected in a fused quartz tube reactor [62]. On CeO_2_ surfaces, MeOH forms the species t-OCH_3_ and b-OCH_3_. When 2-CP hits the surface, a species resembling 2-picolinamide (2-PA) is adsorbed (Figure 5a). MeOH and 2-CP have been shown to compete with one another for access to CeO_2_′s surface areas. ATR-IR spectroscopy reveals that as the process progresses, the 2-PA population on the ceria increases by consuming invader methoxy-forming sites (Figure 5b). The growth of t-OCH_3_ species is prevented by a 2-PA-like species, and the ceria catalyst gradually loses its activation.

### 2.5. Cu-Based Nanoparticle Catalysts

Due to their unusual physicochemical characteristics [63], in a variety of catalytic reactions, including the production of DMC by MeOH-oxidative carbonation [64], carbonate materials that support Cu nanoparticles have drawn a lot of attention in recent years [65,66,67,68,69]. It is well-known that the distribution of active metal species and particle size significantly affect catalytic effectiveness [70]. The particle size and distribution of Cu species on the carbon support have been successfully controlled using a variety of approaches, including modification of the preparation procedure [71,72] and alloying Cu with other metals [73,74,75].

A novel efficient oxidative carbonation catalyst for MeOH was studied by Ren et al. [76]. Here, we describe the preparation of new well-dispersed Cu catalysts supported on carbon microspheres (CMs) using precursor materials, such as Cu(NO_3_)_2_ and cationic sulfonic acid-grouped resins. Additionally, Cu/CMs catalysts were created by subjecting binary precursors to a series of procedures, including carbonization, ion exchange and H_2_ reduction. As Cu^2+^ ions anchor to sites, the functional surface groups are advantageous for improving dispersion and preventing the Cu particles from aggregating/strengthening their catalytic potential. The calcination temperature had a significant impact on both the structural characteristics and the Cu/CM catalyst’s effectiveness. Zhang and colleagues [73] produced Cu species with an average diameter of 12 nm by synthesizing a Cu catalyst supported on activated carbon (AC) modified with HNO_3_. It is possible to visualize the ion exchange reaction between the resin’s cation in the liquid medium (*Cu_sol_^2+^*) and the resin’s cation (*Na_exch_^+^*) as follows:

(9) $$2Na_{exch^{+}}+Cu_{sol^{2+}}=2Na_{sol^{+}}+Cu_{exch^{2+}}$$

**Figure 6:**
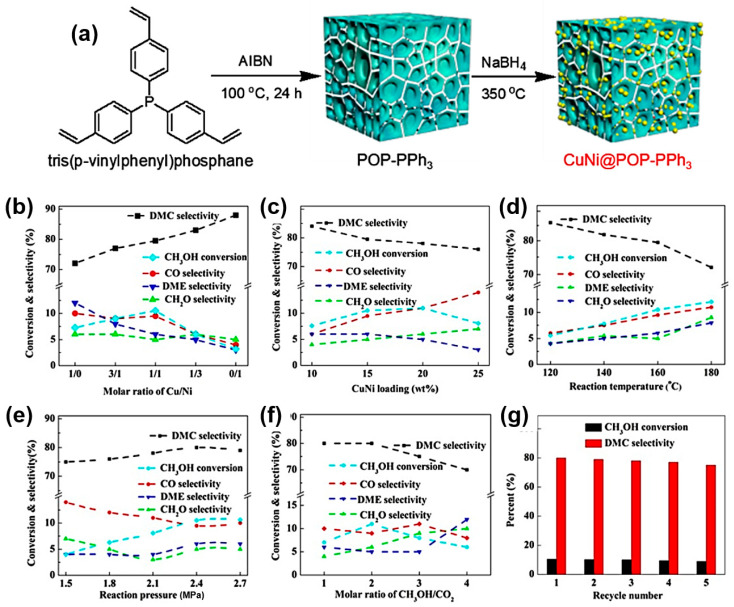
(**a**) Pathway of the synthesis of Cu_x_Ni_y_@POP-PPh_3_. Catalytic performance of the Cu_x_Ni_y_@POP-PPh_3_ as a function of (**b**) Cu/Ni ratio, (**c**) metal loading, (**d**) temperature, (**e**) pressure, (**f**) CH_3_OH/CO_2_ ratio and (**g**) recyclability. Reproduced with permission from Ref. [77]. Copyright 2019 Elsevier B.V.

Li’s group has achieved the effective synthesis of DMC over porous organic polymers (POP-PPh_3_) with a high surface area (Figure 6a) [77]. Through the solvothermal polymerization, Cu–Ni alloy NPs were anchored on the mesoporous structure of the POP-PPh_3_ substance. In order to construct a monolithic Cu_x_Ni_y_@POP-PPh_3_ catalyst, the catalyst was also coated onto a honeycomb ceramic, adopting a new coating technique. The green DMC synthesis uses these monolithic catalysts, which display exceptional catalytic activity.

The impact of reaction conditions on Cu_x_Ni_y_@POP-catalytic PPh_3′_s behavior is summarized in Figure 6. The Cu_x_Ni_y_@POP-PPh_3_ catalyst’s catalytic performance was assessed at 160 °C and 2.4 MPa (15% metal loading). While Ni@POP-PPh_3_ catalysts only showed a marginal conversion rate, Cu@POP-PPh_3_ demonstrated a considerable rate of conversion (8.2% MeOH). In the one-step DMC synthesis, Cu was shown to be more efficient than Ni. The unusual volcano-type activity behavior displayed by Cu_x_Ni_y_@POP-PPh_3_ catalysts (Figure 6b) suggests a considerable synergy between Cu and Ni. It is interesting to note that the nickel species improved the DMC selectivity, as seen when Ni concentrations were increased from 78% to 88% over Cu@POP-PPh_3_. The Cu–Ni alloy loading was raised from 10% to 20% for the Cu_1_Ni_1_@POP-PPh_3_, which allowed for the investigation of the optimal loading of active components (Figure 6c–f). However, it started to decline at a particle loading of 25%, mostly due to the pore-blocking of POP-PPh_3_ at extreme alloy stuffing and the agglomeration of metal particles.

The catalytic cycle suggested for the green DMC synthesis using composites made of Cu–Ni and graphite is presented [78]. The catalytic cycle consists of three main steps: the production of CH_3_O on the metal surface by activating CH_3_OH, the production of CO on the metal surface by activating CO_2_ and the reactions between the CH_3_O and CO species that result in the production of DMC and the regeneration of the metal sites. When reactants are activated and DMC is formed, electron transport can be crucial. The choice of a suitable substrate with remarkable qualities, such as easy electron transport, is likely to favor the reaction [78].

After heating the utilized Cu_1_Ni_1_@POP-PPh_3_ catalyst for 3 h at 350 °C and in the presence of H_2_, it could be used again. As demonstrated in Figure 6g, the Cu_1_Ni_1_@POP-PPh_3_ provided similar catalytic activity throughout the course of five cycles, whereas the DMC selectivity steadily declined from 80% (first run) to 74% (fifth run). According to the oxidation state, Cu^2+^ > Cu^+^ > Cu^0^ [73,79,80], the oxygenated groups on the surface of AC, which supports the Cu catalysts, enhance the Cu dispersion.

In the synthesis of green DMC, copper nanoparticles on different carbon-based substrates, including starch-derived carbon, mesoporous carbon that has been organized, hollow carbon spheres and grapheme [81], showed good catalytic activity. Using DFT calculations, Ren et al. [82] recently examined individual Cu atoms embedded in monovacancy graphene (Cu/MG) and suggested that it is particularly active in the synthesis of DMC. Due to its distinctive qualities, such as its high surface area and strong conductivity, graphene, a 2D carbon material with sp2-bonds organized in the hexagonal lattice, is frequently utilized in electro-catalysis [83,84,85]. Chemical heteroatom doping is an effective way to change the characteristics of graphene among the numerous ways [86]. In comparison to carbon, nitrogen contains an additional electron, which can improve the electrical properties and catalytic abilities of graphene. There have been many doping techniques described, including thermal polymerization, chemical vapor deposition (CVD), plasma spraying [87,88] and others. Depending on their chemical makeup, different nitrogen species evolve at different temperatures. At low treatment temperatures, nitrogen can be added to the graphene substrate in the form of amino-, pyrrolic- and pyridinic-N, while graphitic-N species predominate at higher temperatures, according to the literature [89,90]. Using DFT and experimental research, several nitrogen species have been fully examined with regard to their function in the catalytic efficiency of graphene-based catalysts [91,92,93].

Ren et al. [79] produced DMC over individual Cu atoms embedded in N-doped graphene in response to the aforementioned observations. The catalysts’ structures are based on Cu atoms anchored on graphene, namely graphitic-N-doped graphene (Cu/GNG), pyridinic-doped graphene (Cu/PNG) and amino-doped graphene (Cu/ANG). DMC is produced by activities taking place in the oxidative CBN of MeOH using a Cu/NG catalyst. The calculated binding energies of Cu/PNG, Cu/ANG and Cu/GNG are 449.3, 615.4 and 655.0 kJ mol^−1^, respectively. These values are higher than the calculated binding energy of pure graphene-supported Cu (229.4 kJ mol^−1^ for Cu/PG), showing that N species can effectively fix single Cu atoms, which can improve the catalytic activity in DMC synthesis.

Additionally, during the synthesis of DMC, CO insertion into methoxide is preferred to CO insertion into di-methoxide. With corresponding reaction energies of 63.4, 44.6, 52.1 and 38.2 kJ mol^−1^, CO insertion into methoxide on Cu/ANG, Cu/MG, Cu/GNG and Cu/PNG must pass energy barriers of 92.0, 73.5, 52.1 and 31.0 kJ mol^−1^, respectively. According to these findings, the catalytic activity declines in the following order: Cu/PNG > Cu/GNG > Cu/MG > Cu/ANG. Since it speeds up the synthesis of DMC, the presence of graphitic- and pyridinic-N on the Cu1/NG surfaces is favorable. Additionally, compared to Cu4 clusters and Cu(111) surfaces, Cu/PNG and Cu/GNG had much higher catalytic activity. These results imply that both the attachment sites for individual Cu atoms and the catalytically active sites for the synthesis of DMC are efficiently facilitated by N-doped graphene.

In 2020, Kongkachuichay et al. [94] claimed that the green synthesis of DMC was facilitated by the dispersion of Cu–Ni metals on graphene. Cu/Ni loading on the graphene aerogel was adjusted to 5–20% (equimolar Cu/Ni) concentrations. The 15% CuNi/graphene aerogel catalyst had the highest catalytic efficiency when compared to other loading percentages. Intriguingly, compared to a one-step loading of the Cu/Ni graphene catalyst, a two-step loading procedure boosted MeOH conversion by 18.5% and the DMC yield by 25%. This is because loading Cu–Ni metals during the hydrothermal process avoids Cu–Ni metals agglomeration.

## 3. Conclusions, Challenges and Future Perspectives

Due to their wide range of applications as chemical feedstock, fuel additives, solvents and electrolytes, organic carbonates play a crucial role in creating safe and ecologically friendly chemical processes. The main barrier to the industrial application of organic carbonates is their high cost, which can be significantly decreased by CO_2_, a reactant with a high cost-benefit. DMC has a strong potential as an electrochemical fuel additive to replace hazardous substances because of its desired properties, including good mixing ability, high oxygen content, transparent structure, non-hazardous composition and ease of handling. For DMC synthesis, a number of new approaches has been devised to get around the complexity and limitations of conventional techniques. One of these is the green synthesis of DMC from CH_3_OH and CO_2_, which uses cheap, plentiful feedstocks. This route is strongly constrained by thermodynamic constraints as well as the challenge of activating CO_2_. Thus, the low yield and reaction rate remain a fundamental issue with the direct synthesis of DMC. Finding efficient CO_2_ catalysts and adequate water removal methods has proven to be difficult. There has been a lot of work put into creating new catalysts, including ZrO_2_ and CeO_2_, which show promising catalytic activity and selectivity, but these catalysts can only be employed effectively in conjunction with the right dehydration agents, such as molecular sieves, nitriles, epoxides or ketals. To finally scale up the direct DMC synthesis from CO_2_ and methanol, new, more effective catalysts and complex reaction engineering techniques are required, Table 1. Future industrial demands for the green DMC synthesis may be satisfied by monolithic catalysts coupled with membrane reactors, where the necessary DMC and the water by-products may be efficiently segregated in real-time to increase the DMC yield.

## Figures and Tables

**Table 1:** Summary of reaction conditions and catalytic performances in the direct green DMC synthesis.

Entry	Catalyst	T (°C)	P (bar)	MeOH Conv. (%)	DMC Sel. (%)	Ref.
1	Ti_0.04_Ce_0.96_O_2_	140	22	5.4	83.1	[11]
2	Cu-KF/MgSiO_x_	140	4	9.2	96.0	[25]
3	Zn_0.10_Ce_0.90_O_2_	160	24	20.5	82.1	[50]
4	Bi_0.12_Ce_0.88_O_δ_	140	24	20.6	85.1	[53]
5	CeO_2_-spindles	150	50	63	97	[54]
6	CuNi@POP-PPh_3_	160	24	10.5	80	[77]
7	CuNi/graphite	100	12	10.1	88.0	[78]
8	Zr_0.10_Ce_0.90_O_2_	140	75	11.2	9.6	[95]
9	CeO_2_-4A	120	6	4.0	81.4	[96]
10	Ti_0.10_Ce_0.90_O_2_	140	24	24.3	78.5	[97]

## Abbreviations

DMC	dimethyl carbonate
TFA	trifluoroacetic acid
MTBE	methyl tertbutyl ether
TTF	Dutch Title Transfer Facility
LTA	Linde Type-A
Ov	oxygen vacancies
NRs	nanorods
BTC	1, 3,5-benzenetricarboxylic acid
NPs	nanoparticles
PC	propylene carbonate
DME	dimethyl ether
EPR	electron paramagnetic resonance
TPD	temperature-programmed desorption
XPS	X-ray photoelectron spectroscopy
CH_3_O_a_	adsorbed methoxy
2-PA	2-picolinamide
CMs	carbon microspheres
AC	activated carbon
MG	monovacancy graphene
GNG	graphitic-N-doped graphene
PNG	pyridinic-doped graphene
ANG	amino-doped graphene
POP	porous organic polymer
